# Puerperal Fever in Cook County Hospital

**Published:** 1870-04

**Authors:** 


### PUERPERAL FEVER IN COOK COUNTY HOSPITAL.

MEDICAL EXAMINER:—The following is a report of a committee appointed at the regular meeting of Medical Board of Cook County Hospital, January 31st, 1870, to investigate the causes and management of the recent epidemic of puerperal fever in this hospital, and read at the regular meeting of the Board, February 28th, 1870; and on motion, ordered published in the medical journals of this city, and a copy furnished the Medical Society. T. D. FITCH,

*Secy. Med. Board Cook Co. Hospital.*

TO THE MEDICAL BOARD OF COOK COUNTY HOSPITAL:

*Gentlemen:*—The undersigned, appointed a committee at your last meeting, to investigate the management of the lying-in wards of the hospital, during the prevalence of the late epidemic of puerperal fever, beg leave to report:—That the resources of our art for the prevention and in the presence of such epidemic manifestations may be briefly stated as the following:—

*1st.* Cleanliness of persons, and clothing, and apartments of the lying-in women. Ventilation of the wards; disinfection and removal of any sources of putrid effluvia; avoidance of the results of overcrowding; a properly regulated temperature, and appropriate alimentation.

*2d.* These means failing to prevent the occurrence of cases of the disease, in addition to them, it now becomes necessary to isolate the patients, as promptly and completely as practicable, to make the disinfection and ventilation, if possible, yet more thorough, and on failure of these means, to arrest the disease among the parturient patients.

*3d.* It then becomes necessary to vacate the wards and refuse admission to this class of patients, experience proving that the risk is too great to continue their occupancy.

*4th.* This disuse of the wards should be for such length of time, and seconded by such renovation, cleansing, and disinfection, as experience proves most efficient, in thoroughly removing any real or suspected sources of infection from the wards themselves, the walls, ceiling, floors, etc., and all they may contain of clothing, furniture, etc. The treatment of such cases as do occur, notwithstanding these precautions, is so well settled that it is unnecessary to refer to it in detail; suffice it to say, that the indications to be filled by sedatives, counter-irritants, and opiates, and in toxæmic cases, eliminates with such special modifications as particular complications demand; finding their indications in sound pathology and therapeutics is the practice of the most authoratative names in gynæcological science.

In the light of these principles, your committee has thoroughly investigated the facts in regard to the late epidemic prevalent in the hospital, and find they have been fully and faithfully observed. That scrupulous cleanliness, thorough disinfection, isolation of the patients, so far as practicable, and vacation of the wards were all effected, at such times as were proper and necessary. That the whole house has been thoroughly whitewashed twice, and the lying-in wards three times, since these cases began to appear. That such disinfectants as chloride of lime, carbolic acid, spray of permanganate of potassa, and solution of chlorinated soda have been freely and persistently used. That the methods of treatment put in practice were such as have already been noted, and that in their results the most favorable effects were witnessed, notwithstanding formidable obstacles, which we beg leave to bring to your notice.

It is well known by all familiar with the classes of lying-in patients to be found in a pauper hospital, that they are, from the first, in notoriously unfavorable conditions, for a safe and normal termination of gestation; the immense majority being either the unfortunate victims of seduction, abandoned to hardship and exposure, depressed and debilitated by moral and physical causes, more or less intense and long-continued, or women of abandoned and depraved habits of life, intemperate and exhausted by venereal excesses and diseases, arriving at the hospital as a last resort, having failed of all other means of existence, while it is the exception and not the rule to meet with women in average favorable moral and physical conditions for the act of childbirth. Under these circumstances, if we add to the gravity of this dangerous disease these unfavorable conditions, we should naturally expect a very high mortality, even with the most skilful treatment; which your committee is happy to announce, in this instance, as being much less than could reasonably have been anticipated. In effect, from August, 1869, to February, 1870, the period during which the epidemic influence has principally made itself felt, the number of cases of puerperal fever occurring was 16, of which 10 recovered and 6 died; a lower death-rate than can be shown under like conditions in any published report with which your committee is familiar. And your committee would further and finally report, after careful investigation, that any representations of neglect or mismanagement of this department of the hospital can owe their origin only to one of two circumstances, *viz.:* ignorance of the facts or their wilful falsification. All of which is respectfully submitted.

THOS. BEVAN, M.D., *Chairman,*

W. H. BYFORD, M.D.,

EDWIN POWELL, M.D.

